# Synthetic, Population-Based Virtual Patient Database Using a Digital Twin of the Cardiovascular System

**DOI:** 10.1007/s13239-026-00822-4

**Published:** 2026-02-12

**Authors:** Richárd Wéber, Márta Viharos, Benjamin Csippa, Dániel Gyürki, György Paál

**Affiliations:** Department of Hydrodynamic Systems, Faculty of Mechanical Engineering, Budapest University of Technology and Economics, Müegyetem rkp. 3, Budapest, 1111 Hungary

**Keywords:** Hemodynamics, Virtual patient database, First blood, Demography, Digital twin

## Abstract

**Purpose:**

The goal is to develop a cardiovascular virtual patient database (VPD) combining physiological and demographic data to provide the foundation for future applications in medical diagnostics, decision-making, credibility testing, and formal uncertainty analysis, and to enable its integration with three-dimensional (3D) hemodynamic models and to train neural networks.

**Methods:**

We generate an initial VPD by treating input parameters of a low-dimensional cardiovascular model as stochastic variables. Literature data and sensitivity analysis ensured physiological plausibility, while resampling improved physiological accuracy. Key physiological quantities are included such as systolic and diastolic aortic pressure, radial and carotid pressure, cardiac output, and diagnostic pulse wave velocities. Demographic factors (sex and age) are assigned based on their physiological impact. The open-source hemodynamic solver, first_blood, ensures accuracy and low computational time.

**Results:**

The initial VPD consists of 50,000 Virtual Patients; after resampling, 34,347 remain in the final VPD. The difference of diastolic and systolic aortic pressures between the VPD ($70.24\pm 14.3$ and $116.8\pm 16.11$ mmHg) and the literature ($75.6\pm 12.7$ and $113.0\pm 11.2$ mmHg) is low. The differences caused by the sex of the patient are reproduced well by the VPD: increased diastolic aortic pressure for males ($72.1\pm 12.6$ and $68.5\pm 15.4$ for males and females respectively). The VPD also accurately includes higher pulse wave velocities with age, patients below year 30 have $6.3\pm 0.5$ and above 70 have $8.8\pm 1.2$ m/s with a linear increment in-between.

**Conclusions:**

The proposed methodology and first_blood solver effectively generate physiologically realistic virtual patient waveforms and demographic variability, providing a robust database for 3D cardiovascular simulations, machine-learning training datasets, and potential clinical decision support applications

## Introduction

Digital twins (DTs) in health care can support medical diagnostics and decision-making to prevent or treat cardiovascular diseases. Besides investigating local abnormalities in three dimensions (e.g., aneurysms [1–3]), analysing the entire arterial circulatory system is possible by applying low-dimensional models in zero (0D) or one (1D) dimensions [4]. Although the former can model the distribution of various quantities, such as shear stress, in detail, the latter has the advantage of reduced computational time [5, 6]. Hundreds of simulations can be carried out within hours, utilising the favourable runtime.

A database containing multiple instances of DTs is referred to as a virtual patient database (VPD). By creating thousands of synthetic DTs, different diseases and syndromes can be analysed in detail without the need to collect new, expensive data from a real population, while still relying on valuable population-level information reported in previous studies for model validation and interpretation [7].

A VPD can support the analysis of cardiovascular problems [8]. For example, a VPD modelling the abdominal aorta with both healthy and unhealthy patients could help develop a machine learning algorithm to detect aneurysms using only pressure signals [9]. Different pulse wave velocity (PWV) definitions were analysed using a VPD of 3,325 members to determine which one estimates aortic stiffness more accurately [10]. The same VPD was also used for the computational assessment of the performance of a mathematical algorithm for model-based wave separation analysis [11]. Neural networks can be trained to approximate multiple physiological quantities from wearable photoplethysmography sensors [12, 13], and machine learning algorithms can predict carotid–femoral PWV from radial pressure waves using a VPD [14].

In this study, a synthetic Virtual Patient Database (VPD) is generated for 0D and 1D cardiovascular models, in which the model parameters–such as peripheral resistances or arterial wall elasticities–are treated as probabilistic variables and sampled accordingly. Since such low-dimensional models may include hundreds of parameters, a crucial question is which parameters should be varied and which probability distributions should be assigned to them to ensure physiologically plausible quantities of interest (QOIs).

This paper aims at generating an extensive VPD containing thousands of synthetic DTs of the cardiovascular network. Unlike previous VPDs that are restricted to 0D formulations and therefore neglect essential 1D pulse wave transmission phenomena [8], our database incorporates physiologically relevant 1D hemodynamic features. Furthermore, while many existing approaches rely on individual measurement data as inputs [9–11], which often require ethical approval and limit scalability, our framework is designed to be independent of direct patient-specific measurements. Instead, measurement or simulated data available in the literature are utilized to ensure the physiological validity of the generated DTs. Another important limitation of earlier VPDs is that demographic characteristics such as age and sex are either absent or only partially represented [9, 10], reducing their relevance for population-based studies. In contrast, our VPD systematically assigns age and sex attributes with physiological plausibility to most DTs, enabling investigations into demographic and statistical variability.

To the best of our knowledge, no existing VPD integrates all three aspects–1D hemodynamic modelling, synthetic demographic variability, and independence from directly measured values–within a single framework. The resulting dataset not only enhances physiological realism but also broadens the applicability of VPDs for hypothesis testing, risk stratification, and in silico clinical trials. Looking ahead, the VPD can be employed to evaluate potential treatment strategies in personalized decision-support systems. Moreover, it enables the derivation of boundary condition distributions for 3D treatment analyses–such as flow rate and pressure waveforms–directly from the ensemble of DTs, thereby linking demographic and physiological variability to clinically relevant simulations.

## Materials and Methods

### Modelling the Cardiovascular Network

**Fig. 1:**
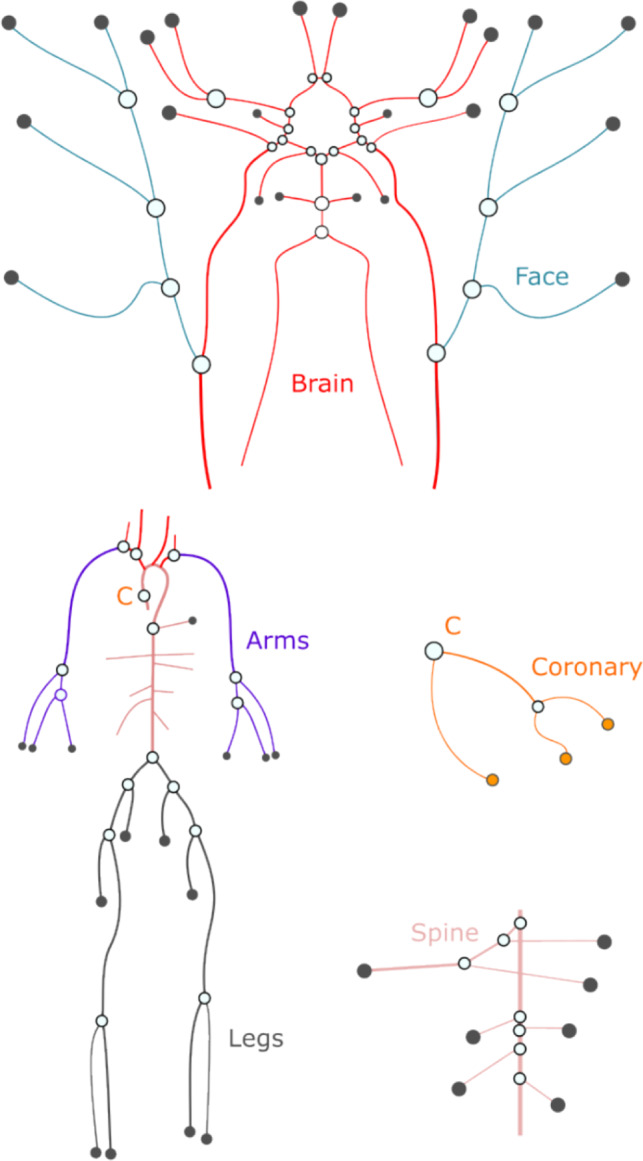
DT of the arterial human circulation with detailed cerebral arteries. Each body location indicated with different colours have the same proportional factor for a given type of variable: *D* for diameter, *l* for length, $R_{perif}$ peripheral and $R_{node}$ for nodal resistance. The Olufsen model factors ($k_1$, $k_2$, $k_3$) are the same for the whole 1D model [20]

The mathematical background of the hemodynamic solver and the hemodynamic model has been presented in detail in previous publications [15, 16]; thus, it is introduced here only briefly. While lumped (0D) models describe the heart, the peripheral circulation, the pulmonary circulation, and the venous system, the arterial network is resolved in 1D (see Fig. 1). Ideal diodes mimic the behaviour of the mitral and aortic valves, and the elastance function models the contraction and relaxation of the heart muscle [17–19]. Although the material properties of the vessel walls are viscoelastic–meaning that both instantaneous and delayed elastic deformations can be observed–the latter effect is negligible under normal physiological conditions; therefore, the applied Olufsen material model neglects it [20]. (Note that the original paper [15] employed a viscoelastic vessel wall model.) The basic model parameters and topology are adopted from the literature: the cardiac model from [21], and the 1D arterial topology from [4]. The peripheral models asmconsist of four RLC circuits representing the arteriolar, capillary, venular, and venous vessels [22]. Additionally, the model includes the coronary arteries responsible for supplying blood to the heart [4], with coronary flow depending on the pressure in the heart chambers [23].

For a 1D arterial vessel, the traditional fluid dynamics equations–the continuity and momentum equations–are solved, assuming elastic walls. Following conventional approaches in hemodynamics [24, 25], the finite-difference MacCormack method is applied to solve the system of equations at the interior points of the vessels. The method of characteristics is then employed to convert the modified mass balance equation and the equation of motion into ordinary differential equations, which are subsequently used to establish the boundary conditions. The 0D models are treated as boundary conditions and are solved using Newton’s method, a widely used technique for handling nonlinear algebraic systems [15].

### Selecting Independent Input Parameters

Most low-dimensional models contain hundreds of parameters. Although reducing the number of arteries can preserve model accuracy [26], this study groups vessels, based on their type and anatomical location in the model (see Fig. 1). The goal of determining the independent parameters is to reduce the problem size without introducing unjustified constraints. First, the peripheral resistances ($R_{perif}$), nodal resistances ($R_{node}$) modelling the flow in smaller, neglected arteries, vessel diameters (*D*), and vessel lengths (*l*) are grouped by location: arms, legs, spine area, coronaries, brain, and face (see Fig. 1). Each DT parameter is determined by multiplying a reference value by two stochastic variables. As an example, for the vessel diameter:

1 $$\begin{aligned} D_{DT} = (D + D_{bl}) D_{ref}, \end{aligned}$$

where $D_{ref}$ represents reference patient data expressed in units of length, and $D_{bl}$ is a dimensionless stochastic variable specific to each body location *bl*, following a normal distribution with a mean of 0 and a standard deviation equal to 0.15 times the standard deviation of *D*. This formulation accounts for individual variability in body proportions by allowing $D_{bl}$ to vary independently across body locations for each patient. Finally, *D* is a dimensionless scaling factor common to all vessels, with its mean and standard deviation determined from the population data. The same approach is applied to the other parameters, resulting in four independent vessel-related parameters: the *D* factor for diameter, the *l* factor for length, the $R_{perif}$ factor for peripheral resistance, and the $R_{node}$ factor for nodal resistance. Lastly, the Olufsen material model [20] includes three independent parameters ($k_1$, $k_2$, $k_3$), which are location-independent and describe the arterial wall material behaviour. In the Olufsen formulation, these parameters define a global pressure–area relationship that is applied uniformly across the arterial tree; therefore, location-independence is an inherent feature of the model.

**Fig. 2:**
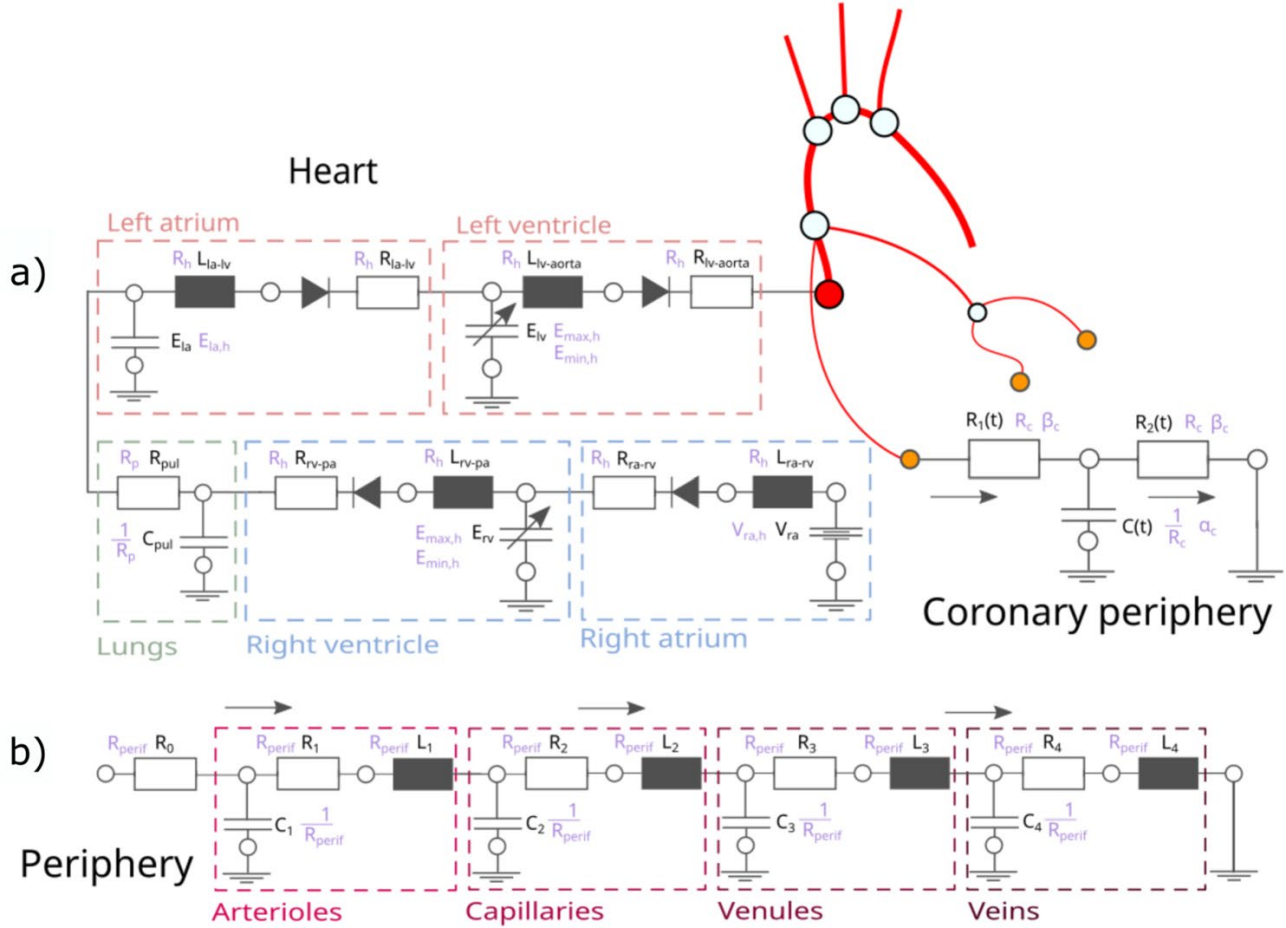
The top side **a** illustrates the left and right side of the heart, and the pulmonary circulation resolved in 0D, the bottom side **b** shows the peripherals of the arterial systems. The black parameters are set for the reference DT, and the purple quantities are the independent factors for the generation of the VPD

Even though the peripheral circulation is modelled using RLC circuit elements (see the bottom panel (b) of Fig. 2), certain considerations apply to the number of independent parameters. First, the time constants of the circuit–either the product of resistance and capacitance or the ratio of inductance to resistance–are kept constant. The second consideration is that the ratios of the resistances are fixed, since each part of the RLC circuit represents a specific type of peripheral vessel, and the ratios of the pressure drops along these vessels are constant [27]. Because of these constraints, there is only one scaling parameter for the peripheral resistances.

The heart, pulmonary circulation, and coronary periphery are modelled using 0D RLC circuit elements (see the top panel (a) of Fig. 2). The heart itself has six independent parameters. $V_{ra,h}$ represents the constant pressure in the right atrium; $E_{max,h}$ and $E_{min,h}$ are the maximum and minimum elastances of both ventricles, respectively; and $E_{la,h}$ denotes the compliance derived from the left atrial volume. $R_h$ and *L* are the resistance and inductance of the heart valves, respectively, and both vary with the same scaling factor. In addition, the heart parameters include the heart rate ($HR_h$), representing the number of cardiac cycles per minute. The pulmonary circulation is characterized by a resistance ($R_{pul}$) and a capacitance ($C_{pul}$), whose scaling factors are reciprocal to keep the time constant of the pulmonary system constant. Finally, the Windkessel models describing the coronary circulation have three independent parameters: one scaling factor for the resistances ($R_c$) and its reciprocal for the capacitance, one parameter characterizing the time-varying resistance ($\alpha _c$), and one for the time-varying capacitance ($\beta _c$) [4]. In total, this results in 6 + 1 + 3 = 10 independent parameters in the 0D elements of the heart, pulmonary, and coronary circulation. Finally, Table 1 lists all unknown model parameters that define the database.

**Table 1:** Summarising all the 17 independent input parameters which characterise a DT

Figures	Location	Type	Notation
Fig. 1	Arteries	Diameter	*D*
		Length	*l*
		Nodal resistance	$R_{node}$
		Material parameters	$k_1$, $k_2$, $k_3$
Fig. 2a	Arterial peripheries	Resistance	$R_{perif}$
Fig. 2b	Heart	Right atrium pressure	$V_{ra,h}$
		Elastance	$E_{min,h}$, $E_{max,h}$
		Left atrium capacitance	$E_{la,h}$
		Resistance	$R_h$
		Heart rate	$HR_h$
	Pulmonary	Resistance	$R_p$
	Coronaries	Resistance	$R_c$
		Time-varying functions	$\alpha _c$, $\beta _c$

Handling these as stochastic variables (normal distribution with a mean and a standard deviation) creates an initial database

### Quantities of Interest

Regarding the physiological QOIs, the radial, aortic, and carotid pressures are considered, including both systolic and diastolic values. In addition, the cardiac output and four diagnostic PWVs (aortic, carotid–femoral, brachial–radial, and femoral–ankle) are included. Table 2 summarizes the literature data for the QOIs used to create the VPD. The diastolic pressure values in the carotid and radial arteries are determined relative to the aortic reference values, as diastolic blood pressure decreases by approximately 1–2 mmHg towards the periphery [28]. Consequently, the target average value for these pressures is set to 2 mmHg lower than that of the aorta, with an assumed standard deviation of 10 mmHg.

**Table 2:** Data for the QOI suggested by literature used for making the VPD

	Parameter	Mean ± STD	References
*p* [mmHg]	Radial diastole	73.6 ± 10	#
	Radial systole	128 ± 20	[29]
	Aorta diastole	75.6 ± 12.7	[30]
	Aorta systole	113.0 ± 11.2	[30]
	Carotid diastole	73.6 ± 10	#
	Carotid systole	116 ± 15.1	[31]
*PWV* [$\frac{{\text {m}}}{{\text {s}}}$]	Aorta	7.63 ± 1.56	[32, 33]
	Carotid-femoral	8.1 ± 1.80	[32]
	Brachial-radial	10.43 ± 1.66	[32]
	Femoral-ankle	9.79 ± 1.78	[32, 34]
*q* [$\frac{{\text {ml}}}{{\text {min}}}$]	Aorta	4570 ± 1090	[32]

*p*: pressure [mmHg]; *PWV*: pulse wave velocity [$\frac{{\text {m}}}{{\text {s}}}$]; *q*: volumetric flow rate [$\frac{{\text {ml}}}{{\text {min}}}$]; STD: standard deviation. The symbol "#" indicates that the values are estimated based on the aorta diastolic pressure

### Creating a Virtual Patient Database

**Fig. 3:**
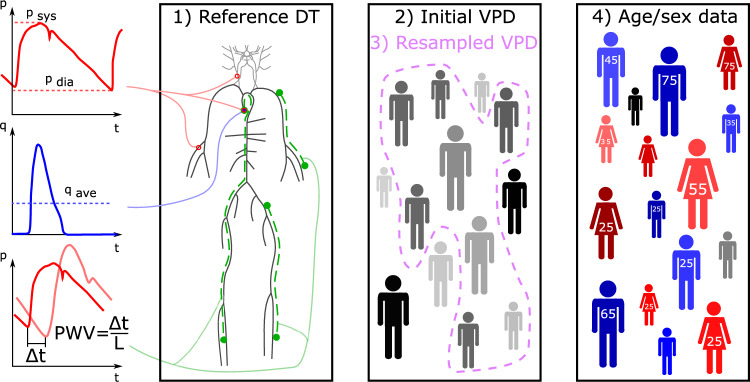
Steps to create the virtual patient database (VPD). The left-side highlights the quantities of interest: diastolic, systolic pressures, cardiac output and pulse wave velocities, then Subplot 1) depicts the layout of an individual DT of the cardiovascular network. Subplot 2) indicates the creation of the initial database and represents the resampling i.e. the removal of outlying DTs. Finally Subplot 4) highlights the assignment of age and sex data.

The method to generate the VPD consists of four main steps. Steps 1–3 in Fig. 3 visualises the concept of the methodology.

1. A reference DT is defined by manual tuning to approximate a patient with average physiological values by setting the average of the input parameters.
2. Simulations sample an initial database by treating the input parameters as stochastic variables, that is, by assigning probability distributions and standard deviations (STDs) to them.
3. Resampling, i.e. deleting outlying DTs, increases physiological sensibility.
4. Finally, sex and age data are assigned to DTs.

#### Initial Database

To create the initial database, the parameters listed in Table 1 need to be defined. First, to establish a representative patient whose QOIs are close to the average values, a sensitivity matrix is calculated to quantify how each input parameter influences the physiological QOIs–that is, how a one percent change in a given input affects a QOI. This information guides a manual, trial-and-error tuning process in which the input parameters are adjusted until the simulated hemodynamic quantities (pressures and cardiac output) approximate the average QOIs. The resulting set of input parameters is then considered to represent the ‘average values’ and is used in subsequent steps.

The second step is to treat the input parameters as stochastic variables and assign probability distributions to each of them. Since the peripheral, nodal, and coronary resistances are expected to have large standard deviations, lognormal distributions are used to avoid negative values, while normal distributions are applied to the remaining parameters. Manual tuning of the reference patient determines the average (mean) values of the parameters. To fully define each distribution, the corresponding standard deviations (STDs) must also be specified. To determine appropriate standard deviations (STDs), we first generate a small test sample of one hundred DTs. Using this sample, the STDs are iteratively adjusted so that the resulting QOIs span the full range of the corresponding literature values. Once suitable STDs are identified, the full initial VPD with $N_0$ DTs is generated.

**Fig. 4:**
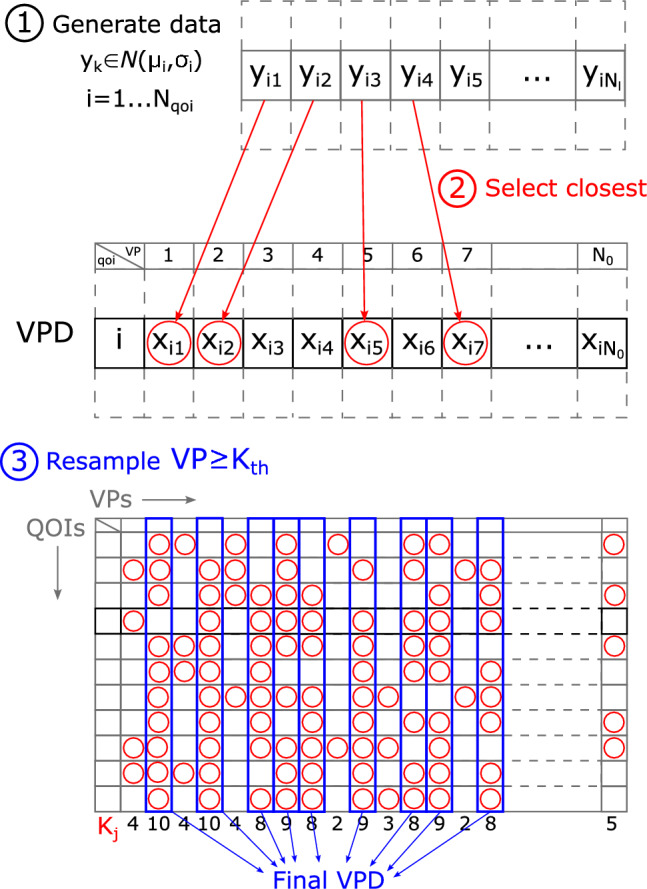
Schematic plan on how to resample the initial VPD and getting rid of physiologically inaccurate VPs. First an artificial dataset is created with desired literature values for each QOI, then the second step matches the closest values within each DT for each QOI. Finally step 3 selects the DTs with the highest number of picked QOI

#### Resampling the Database

The third step is resampling to increase the physiological accuracy of the VPD, compared to the literature data by removing the outlying DTs. The steps are as follows. Follow the steps in Fig. 4.

1. Generate a literature dataset for each QOI with $N_0$ element in each, that is independent of the VPD, with the physiologically desired properties (mean and STD with normal distribution, see Table 2) with chosen $N_l(\le N_0)$ elements. $N_l$ is the maximum size of the resampled VPD. Now for each QOI, there is a literature (or desired) dataset, and calculated DTs in the initial VPD. Step 1) in Fig. 4 highlights a dataset for the i-th QOI.
2. For each QOI and for each element of the literature dataset, select the closest value in the initial VPD respectively. For a given DT, some QOIs will be matched to those in the literature dataset, while others will not, Step 2 indicates one QOI again. The number of selected QOIs is called $K_j$ for the j-th DT.
3. Define a $K_{th}$ threshold value. If $K_j \ge K_{th}$, the j-th DT is resampled to the final VPD, see Step 3) where each column represents a VP, and each row a QOI. Since $N_l \le N_0$, only $K_j (\le N_{qoi}\text {, the number of QOIs})$ number of QOIs of the j-th DT are selected.

A decrease in $N_{l}$ or an increase in $K_{th}$ results in a smaller final VPD. While $N_{l}$ sets the maximum size of the final VPD (that is $N_{vpd}$), $K_{th}$ predicts the accuracy. If $N_{l}$ equals to $N_0$ and $K_{th}=0$, no element is removed from the initial VPD.

Besides examining the histograms of the QOIs in the final VPD, Equation 2 provides a quantitative criterion for selecting suitable values of $N_l$ and $K_{th}$.

2 $$\begin{aligned} F^{rs}=\sum _{i=1}^{N_{qoi}}{\left| \frac{\sigma _{vpd,i}-\sigma _{l,i}}{\sigma _{l,i}}\right| +\left| \frac{\mu _{vpd,i}-\mu _{l,i}}{\mu _{l,i}}\right| } \end{aligned}$$

Here, $\sigma$ denotes the standard deviation and $\mu$ the mean; the subscript *vpd* refers to the simulated values in the VPD, *l* to the literature data, and *rs* to resampling. The value of $F^{rs}$ is a normalized measure that expresses, in a single scalar quantity, how closely the final VPD matches the literature in terms of both mean values and variability. Lower values of $F^{rs}$ indicate a better agreement and are therefore used to evaluate and choose appropriate resampling parameters.

#### Assigning Demographic Data: Sex and Age

Since the literature can be contradictory regarding the sex- and age-based data, a method is defined to determine physiologically acceptable QOI ranges for patients with different sexes and ages. The details of the method can be found in the Appendix, Tables 6 and 7 include the literature QOI values for further target values defined in the Appendix.

The last step is to assign demographic data (sex and age), as shown in Step 4 of Fig. 3, using the determined average values $\mu _{l,ji}^\prime$ and STDs $\sigma _{l,ji}^\prime$ from Tables 6 and 7, where *j* denotes the *j*-th group and *i* the *i*-th QOI. Two bins are defined for sex (male and female) and six for age (–30, 30–40, 40–50, 50–60, 60–70, 70+). In addition, a no-data class containing $N_{nd}$ DTs is included to improve classification accuracy. Although the number of possible divisions is finite–$(2+1)^{N_{vpd}}$ for sex-based and $(6+1)^{N_{vpd}}$ for age-based classifications–examining all possible combinations is computationally infeasible due to the combinatorial explosion. Instead, a two-step procedure is applied. In the first step, each DT is assigned to the class for which the corresponding value of Equation 3 is the smallest, i.e., the group closest to the averages while considering the variances. This allocation primarily ensures that the tendencies of the averages follow the expected trends.

3 $$\begin{aligned} F_j^{DT} = \sum _{i=0}^{N_{qoi}}{\Bigg |\frac{\mu _{l,ji}^\prime -m_{i}}{\sigma _{l,ji}^\prime }\Bigg |} \end{aligned}$$

$m_i$ denotes a QOI for the j-th patient. The resulting grouping serves as a starting point for the optimization. Once this initial allocation is established, the goal is to minimize the value defined by Equation 4, that is,

4 $$\begin{aligned} F^{vpd} = S + \sum _{j=1}^{N_{gr}}\sum _{i=1}^{N_{qoi}}\left( \frac{\mu _{vpd,ji}-\mu _{l,ji}^\prime }{\mu _{l,ji}^\prime }\right) ^2 + \left( \frac{\sigma _{vpd,ji}-\sigma _{l,ji}^\prime }{\sigma _{l,ji}^\prime }\right) ^2, \end{aligned}$$

quantifying how closely a given classification matches the literature values by summing the squared differences between the calculated and literature-based data for both the means and STDs. Here, $N_{gr}$ denotes the number of groups, and *S* is a penalty term proportional to the number of individuals in the no-data class, which should ideally be zero. $\mu _{vpd,ji}$ and $\mu _{l,ji}^\prime$ are the means of the *j*-th group and *i*-th QOI in the VPD and literature datasets, respectively, while $\sigma$ denotes the STD. Starting from the initial state, an iterative procedure is performed: at each step, a DT is reassigned to another class if the change results in a decrease in $F^{vpd}$.

## Results and Discussion

### Virtual Patient Database

An initial VPD of 50, 000 DTs is generated using the manually tuned mean and STD values of the input parameters (see Table 3). Out of the initially generated 50,000 DTs, 14 are removed due to negative input parameters prior to execution. In 678 cases, unhandled issues occurred, including convergence failures and other numerical difficulties. Thus, the initial VPD size $N_0$ equals 49,308. The parameters $N_l$ and *K* of the resampling algorithm are set to 75% of $N_0$ and 8, respectively, in order to achieve favourable histograms for all eleven QOIs and a low value of $F^{rs}$ as defined in Eq. 2. After resampling, 14,961 DTs are removed, resulting in a final database size of $N_{vpd} = 34,347$. The parameter *S* used during sex- and age-based classification is found to yield the most accurate results with values of $N_{nd}/50,000$ and 0, respectively. The total computational time is approximately 2.4 weeks using ten parallel threads on an AMD Ryzen Threadripper 1950X CPU. The evaluation of the hemodynamic simulations dominates the runtime, while the remaining methods require negligible computation time.

**Table 3:** Distributions of the model input parameters

	$R_c$	$\alpha _c$	$\beta _c$	$k_1$	$k_2$	$k_3$
Dist	Lognorm	Norm	Norm	Norm	Norm	Norm
$\mu$	1.00	1.00	1.00	1.14	0.97	1.18
$\sigma$	0.20	0.10	0.10	0.10	0.10	0.35

	$V_{ra,h}$	$R_h$	$E_{max,h}$	$E_{min,h}$	$R_p$	$E_{la,h}$
Dist	Norm	Norm	Norm	Norm	Norm	Norm
$\mu$	1.00	1.00	1.00	1.00	1.00	1.00
$\sigma$	0.10	0.04	0.02	0.02	0.04	0.03

	$HR_h$	$R_{perif}$	*l*	*D*	$R_{node}$
Dist	Norm	Lognorm	Norm	Norm	Lognorm
$\mu$	1.00	1.05	1.00	1.05	1.00
$\sigma$	0.20	0.10	0.06	0.08	0.10

$R_c$ denotes the resistance of the coronary arteries; $\alpha _c$ and $\beta _c$ are parameters of the coronary periphery; $V_{ra,h}$ is the pressure in the right atrium; $R_h$ is the resistance of the heart valves; $E_{max,h}$ and $E_{min,h}$ are the maximum and minimum ventricular elastances, respectively; $R_{p}$ is the resistance of the pulmonary circulation; $E_{la,h}$ is the compliance derived from the left atrial volume; $HR_h$ is the heart rate; $k_1$, $k_2$, and $k_3$ are parameters of the Olufsen material model; $R_{perif}$ is the scaling factor for peripheral resistance; *l* and *D* are the scaling factors for vessel length and diameter, respectively; and $R_{node}$ is the scaling factor for nodal resistance. “Dist.” denotes distribution, “lognorm.” indicates lognormal, and “norm.” indicates normal

**Fig. 5:**
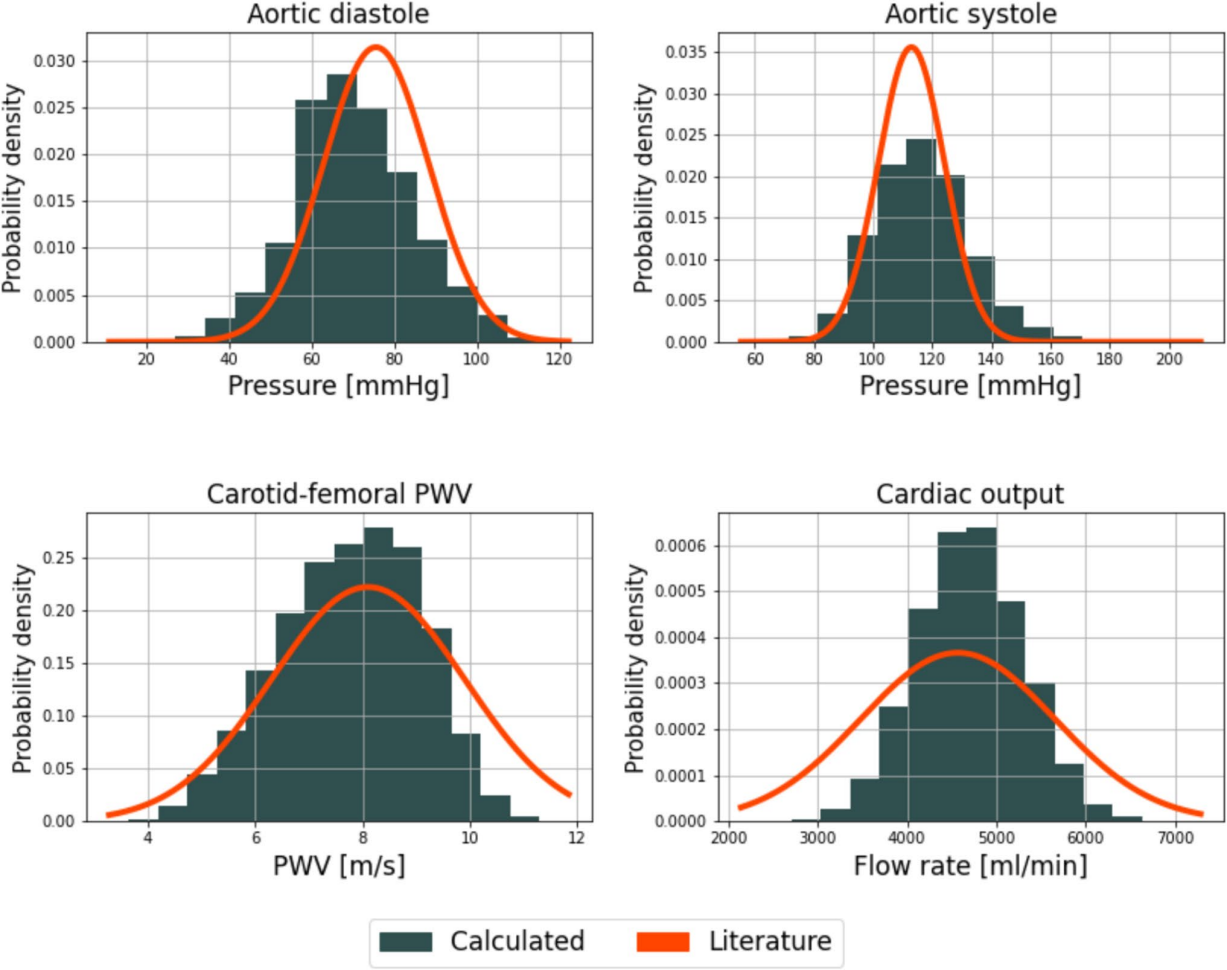
Probability density functions of the final VPD of $N_{vpd}=34,347$, compared to the literature data from Table 1

Figure 5 shows the probability density functions of the QOIs together with normal distributions generated using the literature values from Table 2. The pressure values match the literature well (see the top panel of Fig. 5), although the pulse pressure is slightly higher than reported values. Regarding PWV (see the bottom left panel of Fig. 5), the agreement is high, as the differences from the literature are negligible. Finally, the average cardiac output matches well with the literature (bottom right panel), although its STD is somewhat underestimated. Overall, the quality of the VPD is high, and the resulting physiological values adequately reflect reality. The mean and STD values of the VPD are compared to the reference values in Table 8.

### VPD with Demographic Data

**Fig. 6:**
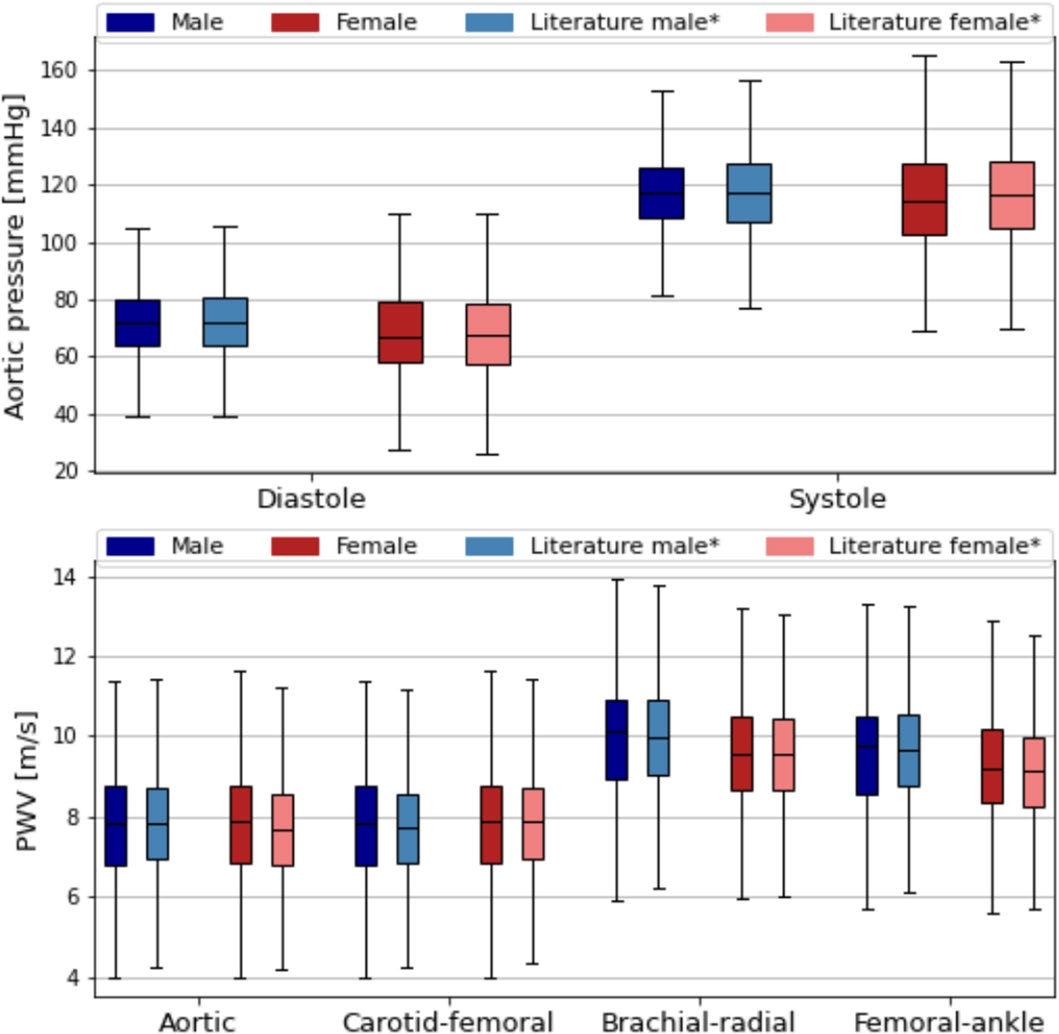
The results of the classification by sex. The aortic pressure and PWV values are compared to the literature data. The symbol “*” refers to the tuned mean and STD values, see the Appendix. The darker colours refer to the VPD, while the brighter ones the literature data

Figure 6 shows the evaluation of sex-based differences compared with literature data, visualised using boxplots. The top panel depicts the diastolic and systolic aortic pressures. Although only small differences between males and females have been reported in large representative populations [30], the VPD captures the main tendencies–namely, decreased diastolic values and increased standard deviations for both systole and diastole in females. The bottom panel shows the PWV values, where the VPD reproduces the higher brachial–radial and femoral–ankle PWV observed in males. These results are compared with the reference values in Table 9.

**Fig. 7:**
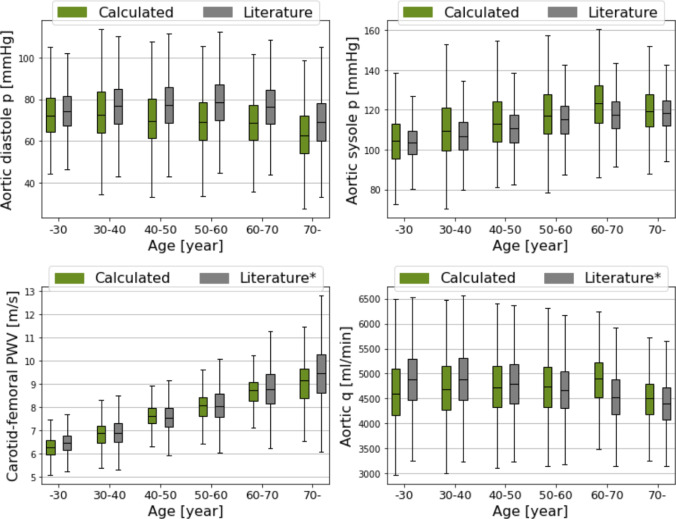
The results of the classification by age. The pressure and PWV values are compared to the literature data. The symbol “*” refers to the calibrated mean and STD values, see the Appendix. The green boxplot includes the VPD data, while the grey ones the literature data

Age-related results are summarised in Fig. 7. The top panel depicts diastolic and systolic aortic pressures as functions of age. The diastolic pressure is slightly underestimated, while the systolic pressure is slightly overestimated. However, the VPD successfully reproduces the decreasing and increasing trends, respectively. The bottom-left panel shows the PWV data: although the standard deviations are underestimated for older age groups, the overall trend is well captured. Finally, the cardiac output is shown in the bottom-right panel. The VPD reproduces the slight decreasing trend with age, although the cardiac output for the 60–70 age group is somewhat overestimated. The corresponding numerical results are presented in Table 10.

**Fig. 8:**
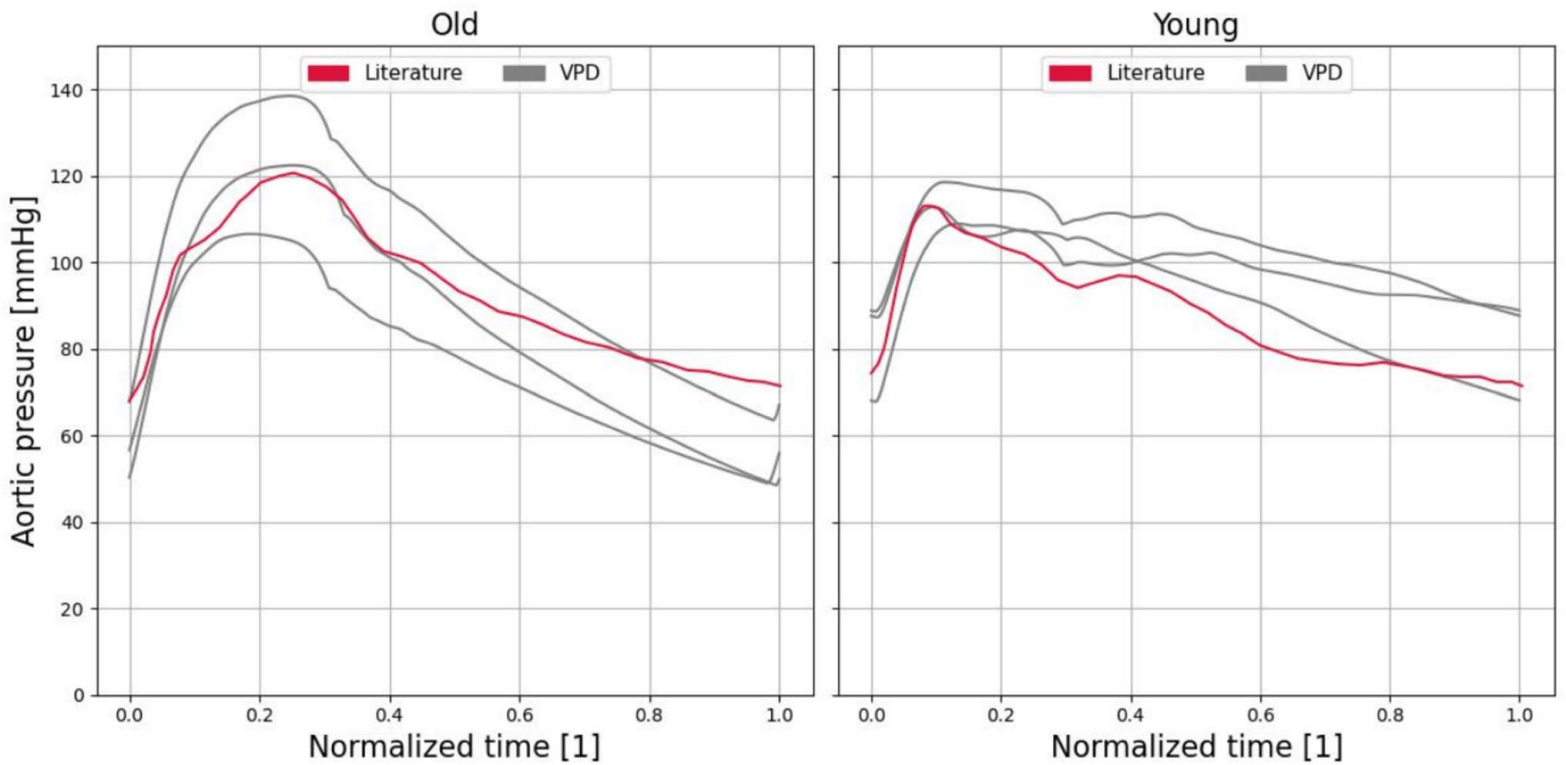
Comparing the aortic pressure waveforms between literature data [35] with black, and three randomly selected VPs with grey for old (left-hand side) and young (right-hand side) patients. The x-axis is normalised time and the signals are shifted to match the periods

In addition to the statistical data, Fig. 8 presents representative waveforms for older patients (left panel) and younger patients (right panel), based on literature data from [35]. In both cases, the qualitative agreement with the literature is strong. Older patients exhibit higher blood pressure values and a more sharply defined systolic peak, whereas the younger cohort displays greater temporal variability and pronounced waviness in the time series.

### Quality and Potential Applications

The quality of the VPD should be interpreted in the context of the accuracy of the underlying medical measurements. While pressure measurements are generally reliable [36], the uncertainty in flow rate measurements is considerably higher. These measurements are typically based on the Doppler effect, which provides the average blood flow velocity. Although velocity measurements already carry a notable error, the uncertainty in the vessel cross-sectional area further amplifies this error [37]. Consequently, the standard deviation of the cardiac output in the VPD slightly underestimates the reference values reported in the literature, which represents a reasonable limitation of the present study. Furthermore, PWV determination relies on simultaneous pressure measurements at two locations; using the measured distance and time delay, the PWV is then calculated. However, the vessel length is often only approximately estimated, and cross-correlation introduces additional uncertainty due to alterations in the pressure waveforms. Overall, the accuracy of the presented VPD is high when considered relative to the measurement uncertainties of the reference parameters.

A potential application of the developed VPD is its integration with 3D simulations to form an in silico framework for clinical decision-support systems. For example, in the subject-specific analysis of a patient with an intracranial aneurysm, parametric inlet and outlet boundary conditions–defined by flow rate and pressure waveforms, respectively–are required for treatment simulations [3]. Stratifying patients by demographic characteristics such as age and sex provides a large set of physiologically meaningful boundary conditions, represented as statistical distributions. Subsequent 3D simulations using Monte Carlo or other sampling-based uncertainty quantification methods can then yield statistical envelopes for predicting treatment outcomes across patient populations.

### Limitations

Determining physiologically accurate QOIs for a population is challenging. Because the reported values originate from different groups of individuals across multiple studies–and may be influenced by factors such as age and vascular disease [32, 38]–some inconsistencies are inevitable. Consequently, when data are collected from different sources, it cannot be guaranteed that they represent the same population. Diastolic blood pressure values between the aorta and peripheral arteries remain relatively constant in a given individual when measured invasively [39]. Therefore, setting significantly different diastolic values as target parameters is not realistic. For this reason, the diastolic pressure averages in the carotid and radial arteries were defined relative to the aortic reference values. Typically, diastolic blood pressure decreases by 1–2 mmHg towards the periphery [28]; thus, the target average values were set 2 mmHg lower than those of the aorta, with an assumed standard deviation of 10 mmHg. This assumption was necessary because the literature sources did not report standard deviations, so the estimate was based on the corresponding aortic value.

Some steps of the methodology remain open to discussion. The definition of the parameter groups is somewhat arbitrary and could be refined or improved. Moreover, although the factor based on body location is intended to introduce variability from homogeneity, the value of 0.15 for its standard deviation is an engineering decision. Further adjustment of this parameter could increase the accuracy of the modelled variability. These factors cannot be considered entirely independent, as they correlate with body height. Additionally, literature data provide ratios of flow rates to specific body regions relative to the aortic flow [40]. Deviating substantially from these values would be undesirable; therefore, peripheral resistances are not treated as fully independent. Treating resistance scaling factors as identical across groups also represents a limitation.

When defining distributions for the input parameters, it is inevitable that some irrelevant virtual patients are included in the preliminary database. The simple reason is that, theoretically, any value can occur in most generated distributions, since the probability density function of the normal distribution is positive for all real numbers. Consequently, removing certain DTs based on the input parameter distributions is necessary. However, the proposed method cannot guarantee the physiological validity of every individual DT; some may still fail to meet physiological plausibility. Future research–possibly employing artificial intelligence techniques such as reinforcement learning–could focus on developing algorithmic approaches capable of assessing the plausibility of each DT individually.

## Conclusions

This paper presents a method for creating a synthetic VPD comprising numerous DTs of the cardiovascular system. Demographic data–specifically sex and age–are assigned to each DT. A hemodynamic solver, called first_blood, solves the one-dimensional conservation of mass and momentum equations and employs the Olufsen model to describe arterial wall behaviour. After defining a reference patient representing an average human through manual tuning, an initial VPD of 50,000 patients is generated by assigning probability distributions to the input parameters. The initial VPD is then resampled using literature data to improve the accuracy of the physiological QOIs, resulting in a final VPD of 34,347 DTs. Sex and age are assigned to the DTs to capture ageing-related changes in QOIs and sex-specific differences. The presented methods require negligible computational time for resampling and classification compared with the runtime of the simulations, which total approximately 2.4 weeks.

The VPD includes six pressure-related QOIs, both diastolic and systolic, which are reproduced well. Although population-wide averages show no major differences between males and females, the standard deviations are higher for women–a trend accurately captured by the VPD. Ageing slightly increases systolic pressure and decreases diastolic pressure, and these effects are also well reproduced. The VPD accurately describes all four analysed PWVs (aortic, carotid–femoral, femoral–ankle, and brachial–radial) across the entire population, as well as within sex and age classifications. Males exhibit higher brachial–radial and femoral–ankle PWVs, and the VPD reflects these tendencies correctly. As PWV values increase with age, the VPD captures this phenomenon effectively, which is essential for future use in decision-support systems for personalised treatment analysis. Despite minor discrepancies in certain quantities, the overall quality and physiological consistency of the presented VPD are high.

## Data Availability

The code of the proposed methods and the original hemodynamic solver, first_blood, are available on GitHub [41].

## Appendix

### Reference Patient Data

Tables 4 and 5 show the reference patient data to ensure the reproducibility of the work. The notations follow Fig. 2. The Olufsen parameters are $k_1=2.0\cdot 10^7\,gs^{-2}cm^{-1}$, $k_2=-22.53\,cm^{-1}$, $k_3=8.65\cdot 10\,gs^{-2}cm^{-1}$ respectively [20]. The arterial network data, such as length and diameters, were initialized from [4], while the 0D element (peripheral RLC circuits and heart-pulmonary network) from [27]. $R_{sum}$ and $R_{0}$ determine $R_1$, $R_2$, $R_3$ and $R_4$ as

$$\begin{aligned} R_1&= 0.65 \, (R_{sum}-R_{0}) \\ R_2&= 0.25 \, (R_{sum}-R_{0}) \\ R_3&= 0.08 \, (R_{sum}-R_{0}) \\ R_4&= 0.02 \, (R_{sum}-R_{0}) \end{aligned}$$

The capacitance and inductance parameters can be calculated as

$$\begin{aligned} C_1 =&\, 0.3394 \, \frac{1}{R_1} \\ C_2 =&\, 0.013052 \, \frac{1}{R_2} \\ C_3 =&\, 0.12441 \, \frac{1}{R_3} \\ C_4 =&\, 0.33 \, \frac{1}{R_4} \\ L_1=&\, 7.4418\cdot 10^{-4} \, R_1 \\ L_2=&\, 3.2539\cdot 10^{-4} \, R_2 \\ L_3=&\, 1.8013\cdot 10^{-3} \, R_3 \\ L_4=&\, 8.0219\cdot 10^{-3} \, R_4 \end{aligned}$$

**Table 4:** Arterial network data of the reference patient

Name	Length *mm*	$$D_{start}$$ mm	$$D_{end}$$ mm	$$R_{0}$$$$mmHg\cdot s / ml$$	$$R_{sum}$$$$mmHg\cdot s / ml$$
Ascending aorta 1	5	29.4	29.3		
Aortic arch A	20	25.1	24		
Brachiocephalic	34	20.2	18		
Subclavian A	34	11.5	9		
Subclavian A	34	11	8.5		
Common carotid	94	13.5	7		
Common carotid	139	12	6		
Vertebral	149	3.7	2.8		
Vertebral	148	3.7	2.8		
Subclavian B - axillary - brachial	422	8.1	4.7		
Subclavian B - axillary - brachial	422	8.1	4.7		
Radial	235	3.7	3.1	21.91	62.24
Radial	235	3.5	2.8	21.91	62.24
Ulnar A	67	3.7	3.4		
Ulnar A	67	4.3	4.3		
Interosseous	79	3	3	60.71	993.8
Interosseous	79	3	3	60.71	993.8
Ulnar B	171	4.1	3.7	15.81	62.24
Ulnar B	171	4.1	3.7	15.81	62.24
Internal carotid	178	5.7	4.3		
Internal carotid	178	5.3	4.1		
External carotid 1	41	5	4.5		
External carotid 1	41	4.7	4.3		
Aortic arch B	39	21.4	20.8		
Thoracic aorta A	52	2	18.9		
Intercostals	80	12.6	9.5	3.10	16.47
Thoracic aorta B	104	16.5	12.9		
Abdominal aorta A	53	12.2	12.2		
Celiac A	20	7.8	6.9		
Celiac B	20	5.2	4.9		
Hepatic	66	5.4	4.4	4.37	42.80
Gastric	71	3.2	3	14.55	63.81
Splenic	63	4.2	3.9	5.65	27.29
Superior mesenteric	59	7.9	7.1	1.84	10.97
Abdominal aorta B	20	11.5	11.3		
Renal	32	5.2	5.2	3.11	13.33
Renal	32	5.2	5.2	3.11	13.33
Abdominal aorta C	20	11.8	11.8		
Abdominal aorta D	106	11.6	11		
Inferior mesenteric	50	4.7	3.2	8.27	81.08
Abdominal aorta E	20	10.8	10.4		
Common iliac	59	7.9	7		
Common iliac	59	7.9	7		
External iliac	144	6.4	6.1		
External iliac	144	6.4	6.1		
Inner iliac	50	4	4	15.80	93.60
Inner iliac	50	4	4	15.80	93.60
Femoral	443	5.2	3.8		
Femoral	443	5.2	3.8		
Deep femoral	126	4	3.7	9.50	56.30
Deep femoral	126	4	3.7	9.50	56.30
Posterior tibial	321	3.1	3	9.50	56.30
Posterior tibial	321	3.1	3	9.50	56.30
Anterior tibial	343	3	2.8	11.12	65.85
Anterior tibial	343	3	2.8	11.12	65.85
Basilar artery 2	20	4	3.6		
Superior cerebellar	10	1.7	1.4	35.08	314.8
Superior cerebellar	10	1.7	1.4	35.08	314.8
Basilar artery 1	5	3.1	2.7		
Post. cerebral 1	2	1.9	1.9		
Post. cerebral 1	2	1.9	1.9		
Post. communicating	4	1.2	1.2		
Post. communicating	4	1.2	1.2		
Post. cerebral 2	59	2	1.8	14.06	126.2
Post. cerebral 2	59	2	1.8	14.06	126.2
ICA distal PCo–ant. chor. seg	2	3.9	3.8		
ICA distal PCo–ant. chor. seg	2	3.9	3.8		
Ant. cerebral 1	12	2.1	2		
Ant. cerebral 1	12	2.1	2		
Middle cerebral M1	8	3	2.8		
Middle cerebral M1	8	3	2.8		
MCA M2 sup. fr. cist. sylvian bif	71	2	1	13.14	117.9
MCA M2 sup. fr. cist. sylvian bif	71	2	1	13.14	117.9
MCA M2 inf. br. dist. sylvian bif	70	2	1	13.14	117.9
MCA M2 inf. br. dist. sylvian bif	70	2	1	13.14	117.9
Ant. cerebral A2	24	1.8	1.7	14.06	126.2
Ant. cerebral A2	24	1.8	1.7	14.06	126.2
Ant. communicating	2	1.3	1.3		
Int. car. sinus	11	4.3	3.9		
Int. car. sinus	11	4.3	3.9		
Ophthalmic	11	1	0.5	35.08	314.8
Ophthalmic	11	1	0.5	35.08	314.8
External carotid 2	61	4	3.5		
External carotid 2	61	4	3.5		
Sup. thy. asc. ph. lyng. fac. occ	101	2	1	55.04	353.8
Sup. thy. asc. ph. lyng. fac. occ	101	2	1	55.04	353.8
Superficial temporal	61	3.2	3		
Superficial temporal	61	3.2	3		
Maxillary	91	2.2	1	45.86	294.8
Maxillary	91	2.2	1	45.86	294.8
Superficial temporal frontal br	100	2.2	1.4	45.86	294.8
Superficial temporal frontal br	100	2.2	1.4	45.86	294.8
Superficial temporal parietal br	101	2.2	1.4	45.86	294.8
Superficial temporal parietal br	101	2.2	1.4	45.86	294.8
Ascending aorta 2	35	29.3	28.8		
Right coronary	53	3.6	2.6		
Left main coronary	5	4.9	4.7		
Left anterior descending	47	3.8	1.5		
Leftc ircumflex	26	3.5	3.1		
Ant. choroidal	36	1.5	1.3	26.27	235.8
Ant. choroidal	36	1.5	1.3	26.27	235.8
ICA distal cnt. chor.–M1 seg	2	3.8	3.8		
ICA distal cnt. chor.–M1 seg	2	3.8	3.8

**Table 5:** Heart and pulmonary circulation data

Component type	Name	Value(s)	Unit
Right atrium			
Battery	$V_{ra}$	6.0	*mmHg*
Inductor	$L_{ra-rv}$	0.375	$mmHg \cdot s^2 /l$
Resistor	$R_{ra-rv}$	7.5	$mmHg \cdot s /l$
Right Ventricle			
Elastance (max, min)	$E_{rv}$	0.5, 0.06	*mmHg*/*ml*
Inductor	$L_{rv-pa}$	0.188	$mmHg \cdot s^2 /l$
Resistor	$R_{rv-pa}$	15	$mmHg \cdot s /l$
Pulmonary artery (P)			
Resistor	$R_{pul}$	90	$mmHg \cdot s /l$
Capacitor	$C_{pul}$	4.0	*ml*/*mmHg*
Left atrium			
Capacitor	$E_{la}$	4.8	*ml*/*mmHg*
Inductor	$L_{la-lv}$	0.3	$mmHg \cdot s^2 /l$
Resistor	$R_{la-lv}$	7.5	$mmHg \cdot s /l$
Left ventricle			
Elastance (max, min)	$E_{lv}$	2.0, 0.06	*mmHg*/*ml*
Inductor	$L_{lv-aorta}$	0.488	$mmHg \cdot s^2 /l$
Resistor	$R_{lv-aorta}$	7.5	$mmHg \cdot s /l$

### Determining the Sex and Age QOI Data

This section presents the method for determining the age- and sex-specific values within the VPD. The algorithms applied in both cases are similar. Tables 6 and 7 summarize the literature data used for classification. Since data from multiple studies are required–and these are sometimes inconsistent–and because the original VPD cannot precisely reproduce all literature-reported QOIs simultaneously, some degree of calibration is inevitable. For example, the average brachial–radial PWV is reported as 10.7 m/s in Ref. [32] and 8.57 m/s in Ref. [34]. Both datasets are necessary, as the former provides age-related data while the latter includes sex-related information. Moreover, the original VPD yields a mean brachial–radial PWV of 9.76 m/s, whereas the literature reports 8.79 m/s for males and 8.36 m/s for females. Mathematically, dividing a sample into two subgroups with lower mean values than the original global mean is not possible. Therefore, the approach aims to preserve the relative tendencies and ratios associated with sex and age rather than the exact absolute values.

The following formulas are applied, where $\mu _{l,ji}$ and $\sigma _{l,ji}$ represent the literature-based mean and standard deviation of the $i^{\text {th}}$ QOI for the $j^{\text {th}}$ class, while $\mu _{r,i}$ and $\sigma _{r,i}$ denote the corresponding mean and standard deviation values from the VPD. *M* indicates the number of groups–two for sex and six for age–and *N* is the number of QOIs (six for sex and seven for age classification). In addition to these main groups, a “no-data” (ND) class is introduced to improve the accuracy of the individual group fits.

Determining the target mean values for the groups involves three steps. The first step is a shift, represented by the term within parentheses in Eq. 5, which gives the signed distance between the average of a given subgroup and that of the overall population. Because the standard deviations $\sigma _{l,i}$ and $\sigma _{r,i}$ generally differ, the ranges of the two datasets also differ (assuming similar distributions). Consequently, the difference between the subgroup and global means is unlikely to match exactly. The second step is a scaling (magnification or reduction) adjustment to account for this difference. The third step is an additional shift, as the first term of Eq. 5 defines the target value relative to the global average.

5 $$\begin{aligned} \mu _{l,ji}^\prime =\left( \mu _{l,ji}-\mu _{l,i}\right) \frac{\sigma _{r,i}}{\sigma _{l,i}}+\mu _{r,i} \end{aligned}$$

6 $$\begin{aligned} \sigma _{l,ji}^\prime =\sqrt{\underbrace{\left( {\sigma _{r,i}}^2-\sigma ^2\left( \mu _{l,i}^\prime \right) \right) M}_{\text {sum of the variances}}\frac{\sigma _{l,ji}^2}{\sum _{k=1}^{M}\sigma _{l,ki}^2}} \end{aligned}$$

he *ji* notation refers to the $i^{\text {th}}$ output variable of the $j^{\text {th}}$ group. After determining the means, it is necessary to calculate the variances. The method described in this section aims to minimise inconsistencies between the total sample variance and the recalculated group-wise averages and variances. The literature provides a straightforward approach for computing the pooled variance of multiple datasets, assuming that the dataset sizes, means, and variances are known [42]. In essence, the pooled variance equals the mean of the within-group variances plus the variance of the group means. When calculating these two terms, both the means and variances must be weighted according to the sizes of the respective groups. By rearranging the equation derived from this relationship, the expression given in Eq. 6 defines the sum of the variances of the subgroups, assuming equal group sizes. Since the goal is to create groups of equal size, the formula simplifies accordingly. The total variance can then be distributed among the groups in various ways; in this approach, the ratio of the subgroup variances is set to match the ratio observed in the reference data.

Let us demonstrate the conversion with a simple example from the database for clarity. Literature mean and standard deviation values used for the gender-based grouping for brachial-radial PWV are denoted with an index of 0 for males and 1 for females: $\mu _{l,0i}=8.79$ $[\frac{{\text {m}}}{{\text {s}}}]$, $\sigma _{l,0i}=1.35$ $[\frac{{\text {m}}}{{\text {s}}}]$, $\mu _{l,1i}=8.36$ $[\frac{{\text {m}}}{{\text {s}}}]$, $\sigma _{l,1i}=1.22$ $[\frac{{\text {m}}}{{\text {s}}}]$, $\mu _{l,i}=8.57$ $[\frac{{\text {m}}}{{\text {s}}}]$, $\sigma _{l,i}=1.30$ $[\frac{{\text {m}}}{{\text {s}}}]$. The VPD mean and standard deviation for brachial-radial PWV are: $\mu _{r,i}=9.76$ $[\frac{{\text {m}}}{{\text {s}}}]$, $\sigma _{r,i}=1.38$ $[\frac{{\text {m}}}{{\text {s}}}]$. The mean and standard deviation values for males can be converted as follows: $\mu _{l,0i}^\prime =\left( 8.79-8.57\right) \frac{1.38}{1.30}+9.76\approx 9.99$ $[\frac{{\text {m}}}{{\text {s}}}]$, $\sigma _{l,0i}^\prime =\sqrt{\left( 1.38^2-\frac{(9.54-\frac{9.99+9.54}{2})^2+(9.99-\frac{9.54+9.99}{2})^2}{2} \right) 2\frac{1.35^2}{1.35^2+1.22^2}}\approx 1.43$ $[\frac{{\text {m}}}{{\text {s}}}]$. Values for the female group can be similarly calculated: $\mu _{l,1i}^\prime \approx 9.54$ $[\frac{{\text {m}}}{{\text {s}}}]$, $\sigma _{l,1i}^\prime \approx 1.29$ $[\frac{{\text {m}}}{{\text {s}}}]$.

**Table 6:** Literature data (mean ± STD) for the QOI used for the classification by sex

	Parameter	Male	Female	Pooled	References
*p* [mmHg]	Aorta diastole	77.6 ± 11.1	73.7 ± 13.7	75.6 ± 12.7	[30]
	Aorta systole	113.2 ± 10.1	112.9 ± 12.2	113.0 ± 11.2	[30]
*PWV* [$\frac{{\text {m}}}{{\text {s}}}$]	Aorta	7.78 ± 1.16	7.64 ± 1.15	7.71 ± 1.16	[34]
	Carotid-femoral	6.31 ± 2.09	6.56 ± 2.09	6.43 ± 2.09	[43]
	Brachial-radial	8.79 ± 1.35	8.36 ± 1.22	8.57 ± 1.30	[34]
	Femoral-ankle	10.09 ± 1.36	9.49 ± 1.3	9.78 ± 1.36	[34]

*p: pressure [mmHg], PWV: pulse wave velocity [$\frac{{\text {m}}}{{\text {s}}}$]

**Table 7:** Literature data (mean ± standard) for the QOI used for the classification by age]

	− 30	30–40	40–50	50–60	60–70	70–	references
Aorta d	74.6 ± 10.5	76.6 ± 12.3	77.4 ± 12.5	78.4 ± 12.7	76.4 ± 12.3	69.4 ± 13.4	[30]
Aorta s	103.6 ± 8.6	107.1 ±10.2	110.9 ± 10.3	115.0 ± 10.1	117.5 ± 9.6	118.5 ± 9.0	[30]
*PWV*							
Aorta	5.9 ± 0.6	6.5 ± 0.8	7.3 ± 0.9	8.0 ± 1.1	8.9 ± 1.3	9.7 ± 1.6	[32]
C-f	6.3 ± 0.7	6.9 ± 0.9	7.8 ± 0.9	8.5 ± 1.1	9.5 ± 1.4	10.4 ± 1.9	[32]
B-r	8.9 ± 0.6	9.5 ± 0.8	10.4 ± 0.8	11.1 ± 1.0	12 1.3	12.8 ± 1.6	[32]
F-b	8.7 ± 0.9	9.2 ± 1.1	10.1 ± 0.8	10.7 ± 1.0	11.6 ± 1.2	12.4 ± 1.5	[32]
*q*							
Aorta	4.88 ± 1.13	4.9 ± 1.13	4.72 ± 1.06	4.52 ± 1.02	4.25 ± 0.95	3.99 ± 0.86	[32]

*p: pressure [mmHg], PWV: pulse wave velocity [$\frac{{\text {m}}}{{\text {s}}}$], q: flow rate [$\frac{{\text {l}}}{{\text {min}}}$]

**Table 8:** Comparing results of the VPD to literature values from Table 2

	QOI	Literature	VPD
*p* [mmHg]	Aorta diastole	75.6 ± 12.7	70.24 ± 14.3
	Aorta systole	113.0 ± 11.2	116.8 ± 16.11
*PWV* [$\frac{{\text {m}}}{{\text {s}}}$]	Carotid-femoral	8.1 ± 1.80	7.77 ± 1.31
*q* [$\frac{{\text {ml}}}{{\text {min}}}$]	Aorta	4570 ± 1090	4709 ± 596

**Table 9:** Comparing results of the VPD to tuned reference values

QOI	Literature*		VPD	
	Male	Female	Male	Female
*p* [mmHg]				
Aorta diastole	72.5 ± 12.6	68.1 ± 15.5	72.1 ± 12.6	68.5 ± 15.4
Aorta systole	117.0 ± 14.6	116.6 ± 17.5	117.9 ± 14.6	115.9 ± 17.5
*PWV* [$\frac{{\text {m}}}{{\text {s}}}$]				
Aortic	7.9 ± 1.3	7.7 ± 1.3	7.8 ± 1.3	7.8 ± 1.3
Carotid-femoral	7.7 ± 1.3	7.9 ± 1.3	7.8 ± 1.3	7.8 ± 1.3
Brachial-radial	10.0 ± 1.4	9.5 ± 1.3	10.0 ± 1.4	9.6 ± 1.3
Femoral-ankle	9.7 ± 1.3	9.11 ± 1.3	9.5 ± 1.3	9.2 ± 1.3

**Table 10:** Comparison between literature data and VPD results for the quantities of interest (mean ± standard deviation)

	QOI	-30		30–40		40–50		50–60		60–70		70-	
		Lit	VPD	Lit	VPD	Lit	VPD	Lit	VPD	Lit	VPD	Lit	VPD
*p*	Aorta d	74.6 ± 10.5	73.2 ± 11.8	76.6 ± 12.3	74.4 ± 13.7	77.4 ± 12.5	71.0 ± 13.6	78.4 ± 12.7	69.5 ± 13.6	76.4 ± 12.3	69.0 ± 13.2	69.4 ± 13.4	63.4 ± 14.4
	Aorta s	103.6 ± 8.6	105.1 ± 12.6	107.1 ± 10.2	111.1 ± 14.8	110.9 ± 10.3	115.2 ± 15.0	115.0 ± 10.1	118.3 ± 14.9	117.5 ± 9.6	123.2 ± 14.0	118.5 ± 9.0	120.4 ± 13.3
*PWV*	C-f	6.5 ± 0.7*	6.3 ± 0.5	6.9 ± 0.9*	6.8 ± 0.6	7.6 ± 0.9*	7.6 ± 0.6	8.1 ± 1.1*	8.0 ± 0.8	8.8 ± 1.4*	8.5 ± 1.0	9.4 ± 1.9*	8.8 ± 1.2
*q*	Aorta	4.88 ± 0.62*	4.65 ± 0.66	4.89 ± 0.*62	4.73 ± 0.62	4.79 ± 0.58*	4.74 ± 0.59	4.68 ± 0.56*	4.72 ± 0.58	4.53 ± 0.52*	4.85 ± 0.54	4.39 ± 0.47*	4.49 ± 0.48

*p*: pressure [mmHg], *PWV*: pulse wave velocity [$\text {m/s}$], *q*: flow rate [$\text {l/min}$]. The symbol “*” refers to the tuned mean and STD values
